# Commandeering Channel Voltage Sensors for Secretion, Cell Turgor, and Volume Control

**DOI:** 10.1016/j.tplants.2016.10.006

**Published:** 2017-01

**Authors:** Rucha Karnik, Sakharam Waghmare, Ben Zhang, Emily Larson, Cécile Lefoulon, Wendy Gonzalez, Michael R. Blatt

**Affiliations:** 1 Laboratory of Plant Physiology and Biophysics, Bower Building, University of Glasgow, Glasgow, G12 8QQ, UK; 2 Centro de Bioinformatica y Simulacion Molecular, Universidad de Talca, Casilla 721, Talca, Chile

**Keywords:** plant cell turgor, volume control, K^+^ channels, voltage-dependent, SNARE protein, Sec1-Munc18 protein, secretion

## Abstract

Control of cell volume and osmolarity is central to cellular homeostasis in all eukaryotes. It lies at the heart of the century-old problem of how plants regulate turgor, mineral and water transport. Plants use strongly electrogenic H^+^-ATPases, and the substantial membrane voltages they foster, to drive solute accumulation and generate turgor pressure for cell expansion. Vesicle traffic adds membrane surface and contributes to wall remodelling as the cell grows. Although a balance between vesicle traffic and ion transport is essential for cell turgor and volume control, the mechanisms coordinating these processes have remained obscure. Recent discoveries have now uncovered interactions between conserved subsets of soluble *N*-ethylmaleimide-sensitive factor attachment protein receptor (SNARE) proteins that drive the final steps in secretory vesicle traffic and ion channels that mediate in inorganic solute uptake. These findings establish the core of molecular links, previously unanticipated, that coordinate cellular homeostasis and cell expansion.

## Trends

Vesicle trafficking (SNARE) proteins ‘commandeer’ the voltage sensor domains of Kv channels to confer a voltage dependence on secretory traffic for coordination with ion transport during cell expansion.

Sec1/Munc18 (SM) protein-mediated regulation of secretion is selective among plasma membrane SNAREs.

SM proteins and Kv channels bind the SNARE SYP121 at overlapping sites, implying a sequential interplay between these proteins to coordinate membrane traffic and transport.

## How Do Plants Regulate Cell Turgor and Volume?

It is crucial for plants that osmolality, hydrostatic pressure, or turgor, and membrane tension are maintained in fine balance. The plant cell is encased by a cell wall, unable to migrate over the body of the organism, unlike the case in animal development, such that plant growth and development are entirely dependent on cell expansion. Cell expansion is also vital for plant movements, including those of stomatal guard cells, which support gas exchange for photosynthesis and control evaporative water loss. With few exceptions, expansion must be coupled with the deposition of new wall material as the cell wall is remodelled and, in all cases, the associated increase in cell volume must be coordinated with secretory traffic that adds new membrane surface as well as extracellular material to remodel the cell wall 1, 2.

Rates of cell expansion must also be matched by solute transport and its accumulation as the cell volume increases. Cell volume affects molecular crowding within the cytosol and intracellular ionic strength 3, 4. If uncompensated, osmotic turgor pressure, which is a primary driver for continued growth, will decline as the cell expands 5, 6. Plant cells synthesise organic solutes and take up substantial quantities of inorganic salts, especially of K^+^, to sustain osmotic pressures against the cell wall. Therefore, from a practical point of view, understanding how ion transport and secretory traffic are integrated in cell growth is bound to address the question that has puzzled plant biologists for over a century: how do plants regulate cell volume and turgor?

Much circumstantial evidence connects membrane traffic with cell volume, osmotic pressure, and ion transport. Decreasing the osmolality outside many plant cells stimulates exocytosis and increases cell volume, while increasing the osmolality outside stimulates endocytosis and a decrease in cell volume 7, 8, 9, 10, 11. The growth of filamentous fungi, root hairs, and pollen tubes are similarly affected by the osmotic gradient across the plasma membrane and affect ion transport activities 5, 6, 12, 13, 14. These and other studies support a model that links cell volume and turgor with membrane traffic and ion transport, but they offer few clues to the underlying mechanisms by which these processes are connected. Here, we revisit the question of how plants coordinate membrane traffic with ion transport, with a focus on recent discoveries that shed new light on these connections.

## A Diversity of Functions for SNARE Proteins in Plants

**Figure 1:**
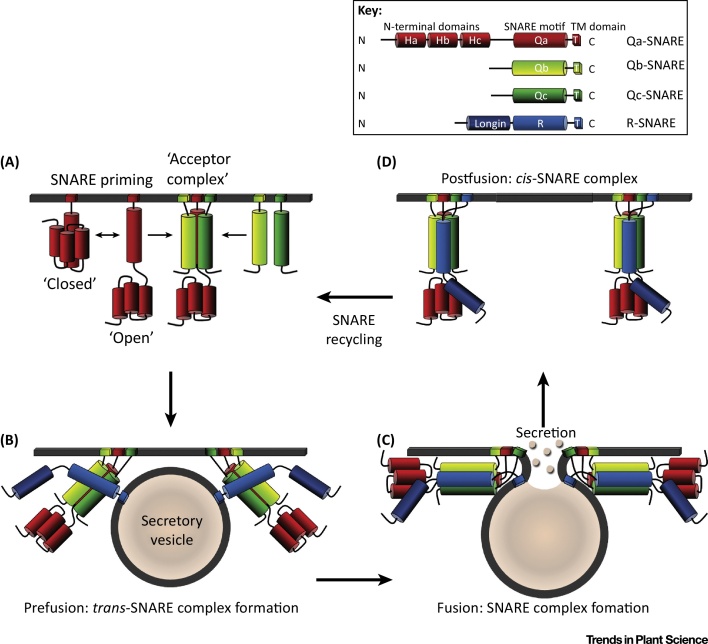
The SNARE Cycle. SNAREs assemble a tetrameric coiled-coil around a central core of three glutamine (Qa, Qb, and Qc) and one arginine (R) residues, in 1:1:1:1 stoichiometry, to draw vesicle and target membranes together for fusion. Each motif is defined by the amino acid at the core of the interacting hydrophobic layers between the respective peptide coils. In some cases, the Qb- and Qc-SNARE motifs reside on a single polypeptide, as is the case for the arabidopsis SNAP33 protein, but, more commonly, each motif in a SNARE complex is contributed by a separate protein 24, 100, 101, 102, 103. (A) Binding of the membrane- and vesicle-localised SNAREs in a cognate SNARE core complex involves priming of the Qa-SNARE at the target membrane. The Qa-SNARE transits from its ‘closed’ to ‘open’ conformation through the unfurling of the Habc domain, which exposes the Qa-SNARE domain for binding with the Qb- and Qc-SNAREs. The Qa-, Qb-, and Qc-SNAREs when bound together form the ‘acceptor’ complex, which is available for binding to the R-SNARE. (B) Formation of the ***trans*****-SNARE complex** following interactions between the R-SNARE on the vesicle and the ‘acceptor’ complex at the target membrane draws the secretory vesicle to the target membrane for fusion. (C) Formation of a stabilised Qa-, Qb-, Qc-, and R-SNARE core complex overcomes the hydration energy barrier of the membrane surfaces and leads to fusion of the secretory vesicle with the target membrane and release of the vesicle cargo. (D) Following vesicle fusion, ***cis*****-SNARE complex** disassembly allows for recycling of the SNARE proteins and sustains further secretory traffic. Abbreviations: SNARE, soluble *N*-ethylmaleimide-sensitive factor attachment protein receptor; TM, transmembrane.

The **SNARE** (see Glossary) superfamily of proteins were first characterised during the 1980s and later recognised as key mediators of secretory traffic in yeast, animals, and plants 15, 16, 17, 18. SNARE proteins incorporate conserved motifs that selectively assemble highly stable, coiled-coil structures to drive membrane fusion (Figure 1). At first glance, plant genomes appear to incorporate substantial redundancy: the arabidopsis (*Arabidopsis thaliana*) genome encodes 54 SNARE proteins compared with the 24 SNAREs of *Saccharomyces cerevisiae* and 36 SNAREs of humans 19, 20, 21, 22. Much gene duplication is thought to reflect a diversification in functions that were necessary for plants to colonise land 23, 24. Plant SNAREs align vesicle traffic with several cellular processes, including gravitropism, pathogen defence, symbiosis, abiotic stress responses, and, of course, cell expansion. Such diversification may relate to the unusual interactions that integrate SNARE activity within the physiological processes they support in plants.

The Qa-SNAREs, or syntaxins, are a case in point. In animals, these proteins lend combinatorial specificity to SNARE interactions for vesicle targeting and secretory membrane traffic, for example as dedicated to neurotransmitter release and insulin-induced GLUT4 traffic 25, 26. In arabidopsis, they have diversified further and engage in specialist roles that differentiate between abiotic and biotic stress responses, and even between responses to bacterial and fungal pathogens. Among the 19 arabidopsis Qa-SNAREs, the nine members of the SYP1 subgroup are evolutionarily related and associate with secretory traffic at the plasma membrane. SYP111 (=KNOLLE), is specialised for homotypic vesicle fusion late in cell division, which leads to the formation of the cell plate [20]; SYP121 (=SYR1/PEN1), SYP122, and SYP132 are expressed throughout the vegetative plant and contribute to abiotic and biotic stress responses 24, 27, 28; and SYP124 and SYP125 are expressed in pollen and are essential for pollen tube growth during fertilisation [29].

Of these Qa-SNAREs, SYP121 and SYP122 are close structural homologues, sharing 64% amino acid identity and 76% similarity across some 310 residues. Initially, these Qa-SNAREs were thought to be functionally redundant. Both are expressed throughout the plant; single mutation of either has little effect on plant growth under many conditions; and growth is greatly reduced only in the *syp121/syp122* double mutant 30, 31. Both SYP121 and SYP122 assemble with the Qbc-SNARE SNAP33 and two near-identical R-SNAREs, VAMP721 and VAMP722, to form SNARE core complexes that drive vesicle fusion and secretion at the plasma membrane 18, 32, 33. However, SYP121 is uniquely important for abiotic stress resistance and stomatal movements; it contributes to the delivery and recycling of K^+^ channels and to long-term photosynthetic capacity under water stress 27, 34, 35; and it may be specialised for fungal pathogen defence [28]. SYP121, and its tobacco (*Nicotiana tabacum*) homologue NtSYR1, were first identified in association with the response to the water stress hormone abscisic acid (ABA) and its regulation of ion channels in the guard cells surrounding the stomatal pore of leaves 34, 36. It was recognised early on that the role of SYP121 in trafficking per se was not sufficient to explain much of the cellular physiology associated with abiotic stress 34, 35, 37. Similarly, VAMP721 and VAMP722 were previously assumed to be functionally redundant, the double *vamp721/vamp722* mutant only exhibiting an embryo-lethal phenotype 29, 38. However, new evidence indicates a specialisation for VAMP721 that aligns with that of SYP121. As we note below, these SNAREs engage directly with a subset of K^+^ channels, and this specialisation is almost certainly a consequence of these interactions.

## Solute Transport, Turgor, and Cell Volume

Plants energise the plasma membrane using H^+^-ATPases to pump H^+^ across the membrane, generating a substantial membrane voltage, inside negative, as well as a pH gradient, inside alkaline. Membrane voltage often comprises some 50% of the electrochemical gradient for H^+^, or Δ*μ*_H_, across the plasma membrane. Voltage is used to drive the transport of other solutes, either together with the pH gradient (for transport coupled to H^+^ return across the membrane) or by itself [39]. K^+^ is the single, most prevalent, inorganic solute in the plant cell. Plants maintain K^+^ concentrations near 100 mM in the cytosol, even when soil K^+^ concentrations are 10^3^- to 10^4^-fold lower and often highly variable 40, 41. K^+^ uptake, along with that of NO_3_^–^ and Cl^–^, is widely recognised to predominate in osmotic solute accumulation for turgor that powers cell expansion in plants. Indeed, in most agricultural soils, nutrient flux is dominated overwhelmingly by K^+^ and NO_3_^−^ 40, 42, 43.

Electrophysiological studies of a variety of plant cells have shown that the dominant transporters – pumps, H^+^-coupled carriers, and channels – are generally present on the plasma membrane. They can be engaged to carry solute across the membrane, but are often kinetically limited by substrate (ion) concentration and, most importantly, by membrane voltage. Voltage controls the K^+^ channels that account for the bulk of K^+^ uptake under most physiological conditions, and it is also a key factor determining ion uptake through other transporters [40]. This situation contrasts with the common misconception that transporters are either present or absent from the membrane in response to various stimuli, including nutrient availability (Box 1). One inference to be drawn from these observations is that K^+^ flux is unlikely ever to be zero. Instead, K^+^ transport is expected to oscillate between uptake and loss over periods of tens of seconds to many minutes. Therefore, dynamic control of solute content is most likely achieved by ‘time-averaging’ net solute flux under control of the membrane voltage. The same applies to the other major osmotic solutes and the interpretation fits closely with observations of voltage and solute flux oscillations 14, 44, 45, 46, 47, with quantitative systems models of transport 44, 48, 49, 50, and with secretion and growth rate in root hairs and pollen, where these processes are easily quantified [14]. In short, membrane hyperpolarisation is essential to engage net solute uptake, its depolarisation facilitates net solute loss, and the balance between these two states of the membrane is connected to cell growth.

Of course, translational and post-translational controls introduce further regulatory controls on solute transport. The voltage range over which many ion channels activate, often referred to as ‘**gating**’, may be affected, for example, by Ca^2+^ binding, nitric oxide, and protein (de-)phosphorylation 48, 51, 52, 53. Voltage-dependent gating is best described in the whole cell by its midpoint voltage (**V**_**1/2**_) at which half-maximal activity (conductance) is achieved. The actions of these several signals may displace V_1/2_ and, hence, the apparent threshold for channel activation, by 20-40 mV or more. Given that the plant cell membrane typically varies across a relatively narrow range of voltages, approximately between –180 mV and –50 mV, such changes in V_1/2_ can have significant effects on the time-averaged net rates of ion flux. Channel activity also depends on the population of K^+^ channels present at the membrane, although, in the first instance, such changes will affect the maximum conductance of the population rather than V_1/2_ 8, 27, 35, 37. Only over longer timescales may translational control alter the distribution between channel subpopulations 54, 55. Even so, these additional controls leave unresolved the most fundamental questions about the mechanisms by which solute uptake is regulated dynamically with membrane traffic as the cell expands.

## SNARE Binding Affects the Voltage Sensitivity of Kv Channels

The first clues to a mechanism coordinating vesicle traffic and ion transport appeared in 2009 with the publication by Honsbein *et al.* [56] of a direct, physical interaction between the Qa-SNARE SYP121 and the **Kv channel** KC1. Subsequent work showed that SYP121 also interacts with a second Kv channel, KAT1, a dominant inward-rectifying K^+^ channel in guard cells and several other cell types [57]. Channel binding in each case was specific for SYP121, but not for SYP122, and binding occurred at the plasma membrane independent of any traffic of the channel to the membrane. KC1 is a close homologue of KAT1, and assembles with another inward-rectifying K^+^ channel subunit, AKT1, as part of a major pathway for K^+^ uptake at the root epidermis [54]. By virtue of its heteromeric assembly, these channels activate only near –200 mV, the voltage extreme of the plant cell, but on interacting with SYP121, the channels activated at voltages approximately 70–100 mV more positive 56, 58. Thus, SYP121 interaction with KC1 is essential to displace V_1/2_ for the KC1-AKT1 channel into the physiological range of voltages normally observed in the root epidermis to facilitate K^+^ uptake. In support of this interpretation, the mutant *syp121*, but not *syp122*, phenocopied the *akt1* and *kc1* mutants, showing little measurable inward-rectifying K^+^ current and failed to grow at submillimolar K^+^ concentrations when channel-mediated K^+^ uptake was limiting.

Subsequent studies [58] resolved the binding site on SYP121 as the linear motif F^9^xRF close to the cytosolic N terminus of the Qa-SNARE and confirmed the ability of SYP121 to complement the *syp121* mutant. Intriguingly, the cognate R-SNARE VAMP721, which is localised to the vesicle membrane before fusion, also binds and modulates the activity of the K^+^ channels. Zhang *et al.* [59] found that VAMP721 binds both the KC1 and KAT1 channel subunits via a site centred around Tyr^57^ within the longin domain of the R-SNARE and separate from the core α-helix that assembles in the SNARE complex. Binding displaced the V_1/2_ for gating to more negative voltages and reduced the maximum conductance, both in K^+^ channels assembled of KC1 and AKT1 subunits and in the KAT1 channels when expressed heterologously. Overexpressing VAMP721 in arabidopsis also suppressed the inward-rectifying K^+^ current of the root epidermis. From the viewpoint of the capacity for K^+^ uptake, these actions effectively counter those of the Qa-SNARE: it is as though channel activity were coordinated in concert with the respective SNARE binding partner, and it begs the question ‘Could K^+^ flux be temporally coupled with a binding exchange between cognate SNAREs during the process of vesicle fusion?’.

## Commandeering Kv Channel Voltage Sensors for Voltage-Sensitive Secretory Traffic

If these findings show that SYP121 and VAMP721 bind in multimeric complexes to control channel-mediated K^+^ uptake, they leave unresolved the consequences for membrane traffic itself. How does SYP121 bind the channels and does channel binding also promote secretory traffic, as it does channel activity? Given the cytosolic location of the F^9^xRF motif of SYP121, it comes as no surprise that the complementary binding site on the Kv channels resides on the cytosolic side of the membrane. However, what was unexpected was the isolation of the critical binding motif RYxxWE to the VSD [60], well removed from the channel pore and adjacent the base of the S1 α-helix. Voltage-gated K^+^ channels of plants (including KC1, AKT1, and KAT1) belong to the superfamily of Kv channels that are found across phyla. Kv channels typically comprise six membrane-spanning α-helices. The first four α-helices (S1–S4) form a semiautonomous voltage-sensor domain (VSD), and the remaining two α-helices (S5 and S6) line the channel pore 61, 62. The VSDs of these channels incorporate a series of fixed positive charges that transduce changes in voltage across the membrane, driving highly conserved conformational changes that move the VSD part-way across the membrane and open the channel pore 61, 63, 64, 65.

The consequences for secretory traffic were equally surprising. Overexpressing the KC1 channel or the VSD alone was sufficient to rescue traffic in the presence of the dominant-negative Qa-SNARE peptides SYP121^ΔC^ and SYP122^ΔC^ (which normally block traffic to the plasma membrane), but only in arabidopsis expressing SYP121. These findings showed that the critical interaction occurs at the plasma membrane with the native Qa-SNARE. Furthermore, VSD expression on its own promoted secretory traffic, but only if the VSD achieved the open channel conformation or could be driven to this conformation by voltage: thus, a mutant VSD that effectively ‘locked’ the voltage sensor in a conformation corresponding to an open channel promoted traffic, whereas a mutant that locked the VSD in the conformation corresponding to the closed channel did not [63]. The findings demonstrate that channel binding facilitates secretory traffic in a voltage-dependent manner. In effect, the Qa-SNARE commandeers the channel VSD to ‘sense’ the voltage bias for transport and adjust the rates of secretory traffic in parallel with solute uptake.

**Figure 2:**
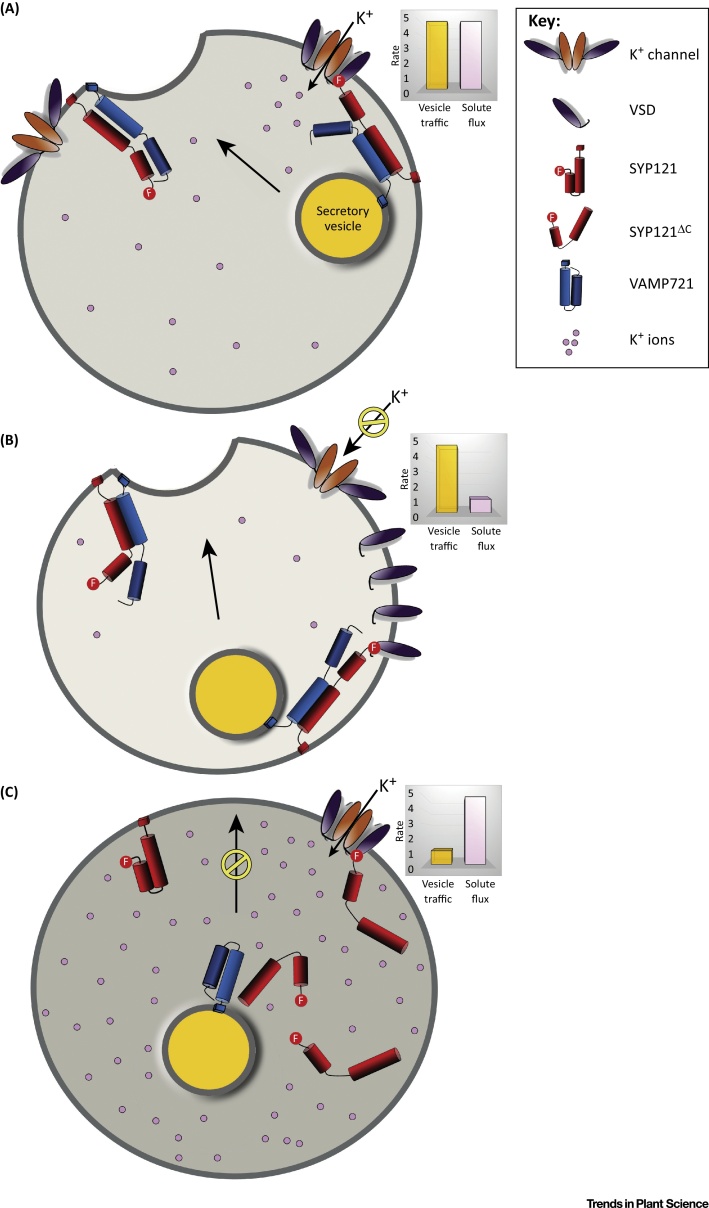
Complementarity of SYP121^ΔC^ and the Kv Channel Voltage-Sensor Domain Is A Predictable Consequence of Their Dual Functionalities. K^+^ channels are shown with orange pore domains and purple voltage-sensor domains (VSDs). SYP121 is shown in red with membrane anchor, longer Qa-SNARE domain, and shorter Habc domain; the dominant-negative SYP121^ΔC^ is shown lacking the membrane anchor; VAMP721 is shown in blue with a membrane anchor, longer R-SNARE domain and a shorter longin domain. The FxRF motif of SYP121 with the VSD is shown as a circle at the SYP121 N terminus. Other SNARE proteins have been omitted for clarity. Comparison of the relative rates for vesicle traffic and solute influx are represented by bar graphs (right). (A) In the absence of either dominant-negative fragment, SNARE binding with the K^+^ channels facilitates the activation and balance of both processes. (B) Overexpressing the channel VSD facilitates SNARE-mediated traffic, but competes with the native channels for SNARE binding, thereby suppressing K^+^ influx and its accumulation. (C) Overexpressing the SYP121^ΔC^ fragment facilitates K^+^ flux by binding and activating the K^+^ channels, but the SYP121^ΔC^ fragment competes with the native SNARE at the plasma membrane for assembly in the SNARE complex needed to drive vesicle fusion. Thus, in each case, the dominant-negative fragment uncouples vesicle traffic and K^+^ uptake with complementary effects on the two processes. Abbreviation: SNARE, soluble *N*-ethylmaleimide-sensitive factor attachment protein receptor.

Such a close juxtaposition of transport with vesicle traffic also underpins the complementary actions of the dominant-negative peptides of the Qa-SNARE and K^+^ channel. Expressing the cytosolic SYP121^ΔC^ peptide was observed in early experiments to block secretory traffic, most likely because the peptide retains the ability to bind its cognate SNARE partners, but is unable to drive vesicle fusion without the C-terminal transmembrane domain 33, 66, 67. Unexpectedly, SYP121^ΔC^ was found also to lead to a substantial rise in cellular K^+^ and osmotic content 59, 67, 68. As noted above, expressing the channel VSD promotes secretory traffic; it also suppresses K^+^ uptake and osmotic content [59]. A simple and overarching explanation lies in the coordinate stimulation of vesicle traffic and ion transport mediated by the interacting partners. SYP121^ΔC^ retains the capacity for K^+^ channel binding [59] and, thereby, for activation of the K^+^ channels; therefore, SYP121^ΔC^ can be expected both to compete with the native SYP121 for cognate SNARE partners, thus blocking traffic, and to promote K^+^ uptake through its binding with the K^+^ channels at the plasma membrane. Similarly, the VSD retains the ability to bind SYP121, but it does not conduct K^+^ across the membrane. So, VSD binding can be expected both to promote vesicle traffic through its interaction with SYP121 and, by competing with the native K^+^ channels for Qa-SNARE binding, to suppress SNARE-mediated stimulation of K^+^ uptake (Figure 2). In short, these experiments elegantly demonstrate that the protein fragments, which retain the corresponding partner-binding domains but only partial functionality, are each able to uncouple secretory traffic from solute transport with predictable and complementary effects.

## A Molecular Governor for the Control of Cell Expansion

In principle, vesicle traffic to and from the plasma membrane may affect the population of ion channels and other transporters, thereby altering the capacity for ion transport. So, why should coordinating vesicle traffic and ion transport directly, independent of such traffic, be so important? During any one day, more than twice the total water content of a plant, along with a substantial quantity of solutes, passes from root to shoot, driven by evaporative demand 69, 70, 71. Variations in water and solute flux over the diurnal cycle may vary rapidly, they affect cellular water potentials, and, consequently, turgor, with direct consequences for cell growth; for example, cell expansion rates in leaves vary in inverse proportion with transpiration and in direct proportion with leaf hydration 70, 72. Clearly, close and dynamic control of membrane transport, solute accumulation, and water flux is essential at the cellular level to integrate these activities with the variations in water and solute availability throughout the hydraulic network of the plant. Most importantly, this control needs to be coupled to vesicle traffic to balance solute transport with the rates at which new membrane surface is added as the cell volume increases.

Direct, mechanical coupling between key components of the vesicle trafficking machinery and a dominant subset of solute transporters is one answer to this need. Such a basal level of control may also be seen as an energy-efficient solution. It does not require a costly overhead in regulatory feedback networks or additional transcription, translation, or protein turnover in SNARE components and membrane transporters. Indeed, in plants, for which voltage comprises a significant fraction of the energetic charge on the cell membrane, simply modulating the population of transporters, such as K^+^ channels, through vesicle traffic and channel turnover is an inefficient way to control the rates of solute uptake. Both experimental evidence and quantitative models have shown that the benefits of altering the number of functional channels at the plasma membrane are offset by compensatory changes in voltage itself 60, 73, 74. Again, these and other studies point to voltage itself as the most efficient point of control for traffic, as it is for ion transport (see the section ‘Solute Transport, Turgor, and Cell Volume’ and Box 1).

In effect, membrane voltage is both a driver for, and is responsive to, all charge-carrying ion transport across the plasma membrane. Membrane hyperpolarisation is usually driven by activation of H^+^-ATPases and is generally important to accelerate solute uptake. Therefore, it makes good biological sense for the plant cell to use voltage as a proxy for transport activity. The voltage proxy is especially important when transport is expected to oscillate with voltage and dynamic control of solute content is achieved by ‘time-averaging’ net flux. Given that channel VSDs respond to transmembrane voltage by undergoing substantial conformational changes, the conformational status of the VSD provides an excellent running measure of voltage with a resolution timeframe of milliseconds. Similar to James Watt's application of the mechanical governor 75, 76, SNARE-VSD binding serves as a molecular governor, a coupling that enables mutual coordination of the two processes so that each facilitates and tempers the activity of the other.

**Figure 3:**
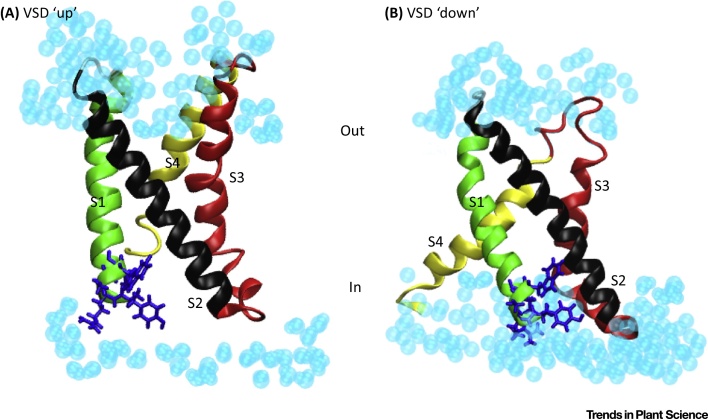
Voltage Alters the Exposure of the Kv Channel Voltage Sensor to the Cytosol. Side views of the voltage sensor domain (VSD) of the KAT1K^+^ channel are shown in the ‘up’ (A) and ‘down’ (B) conformations. For clarity, the membrane bilayer is not shown and only water molecules (light blue) on either side (inside and outside) of the membrane are visible. Transmembrane α-helices are colour coded in green (S1), black (S2), red (S3), and yellow (S4). Residues RYxxWE that form the binding site for SYP121 are included in blue as stick representations. The molecular dynamics of each conformation were modelled using as a template the entire α-subunit of Kv1.2 obtained by the Rosetta method 63, 107. Each model was analysed after embedding within a lipid bilayer in a periodic boundary condition box with water molecules, K^+^ and Cl^–^ ions, and was optimised using energy minimisation and equilibration at 298 K for 5 ns with a harmonic restraint of 0.5 kcal/mol Å^2^ applied to the backbone atom using NAMD [108]. As the membrane is hyperpolarised, movement of the S4 domain within the transmembrane electric field drives the transition to the ‘down’ conformation and is coupled to rotation of the S5 and S6 α-helices to open the channel pore (not shown). Here, the ‘down’ conformation corresponds to the open channel. On the basis of simulations [63], it appears also to promote the aqueous exposure of the RYxxWE motif at the cytosolic surface of the membrane. Images created with VMD [109].

How VSD conformation is coupled to the Qa-SNARE remains a big question. Conformational changes of Kv and other channel VSDs within membranes are known to affect the exposure of significant portions of VSD structure to the cytosolic side of the membrane 61, 62. Thus, with the RYxxWE motif of the channel positioned close to the cytosolic surface of the membrane [59], its availability for SNARE binding may depend on VSD conformation (Figure 3). Conceivably, then, if binding and debinding are sufficiently rapid, SNARE binding may simply report the time-averaged conformation of the VSD. Equally plausible, however, SNARE binding may be retained, VSD conformation being transduced through the bound SNARE to affect vesicle traffic. In either of these scenarios, binding may also prolong the open state of the (inward-rectifying) channel or reduce the time it remains in the closed state, thereby promoting K^+^ influx. Almost certainly, together with these processes, we also need to consider an exchange of channel binding between cognate SNAREs [59] and how this might be coordinated during vesicle fusion.

## Temporal Coordination of Vesicle Traffic with Ion Transport: A Role for Sec1/Munc18 Proteins?

One clue to a possible exchange in channel binding between SNAREs comes from the basic structure of SYP121. Qa-SNAREs, including SYP121, incorporate a C-terminal membrane anchor preceded by a cytosolic α-helical (H3) domain that comprises the Qa-SNARE motif and assembles with cognate SNAREs in the core complex. At the N terminus of the H3 domain are a set of three antiparallel α-helices, the Habc domain, and an unstructured N-terminal sequence of 20–40 residues. The Habc domain folds back on the H3 α-helix to prevent access to that α-helix, rendering the SNARE inactive in a so-called ‘closed’ conformation 21, 24, 77. Priming of the Qa-SNARE involves unfurling of the Habc domain to expose the Qa-SNARE motif in a fusion competent conformation (Figure 1).

For many Qa-SNAREs, passage through this conformational switch depends on binding and debinding of one of the **Sec1/Munc18 (SM)** family of proteins 33, 78. SM proteins form a clothes-peg-shaped structure with a major cleft that binds Qa-SNAREs in the closed (inactive) conformation, stabilising the protein against promiscuous interactions. They also show at least two other modes of binding. One of these modes engages the Qa-SNARE N terminus, binding it within a minor cleft on the SM surface, and the other involves ‘clamping’ of the SM major cleft over the assembled SNARE complex to stabilise it 79, 80. Binding of the Qa-SNARE N terminus within the SM minor cleft is thought to spatially coordinate and facilitate the unfurling of the Qa-SNARE, and it affects the speed of fusion 80, 81, 82. Even so, what drives the conformational switch between binding modes is unclear and is almost certain to depend on ancillary interactions. Recent studies highlighted the binding of Munc13 with the binary Qa-SNARE-SM pair of Syn1A and Munc18 to accelerate SNARE complex assembly and neurotransmitter release [81]. Munc13, and its homolog Unc13 of *Caenorhabditis elegans* [83], are large, Ca^2+^- and phospholipid-binding proteins that have some similarities to proteins of the exocyst complex involved in vesicle tethering before fusion 84, 85, 86.

The arabidopsis SM protein SEC11 (=KEULE) was originally identified as a factor supporting Qa-SNARE SYP111 activity during cell plate formation [87]. SEC11 also binds SYP121 at the plasma membrane and affects its assembly with VAMP721 and SNAP33 as well as secretory traffic [88]. Arabidopsis harbours six proteins with weak homologies to Munc13, but only PATROL1 functions in vesicle traffic in vegetative tissues [89]. The other five gene products are pollen specific 90, 91 or are isolated to chloroplasts and mitochondria [92]. Significantly, PATROL1 affects H^+^-ATPase traffic to the plasma membrane, which is independent of SYP121 and SYP122 [37] and does not affect their associated cargos 24, 37, 89, 93. Thus, PATROL1 targets a trafficking pathway that is distinct from that of SYP121 and SEC11.

**Figure 4:**
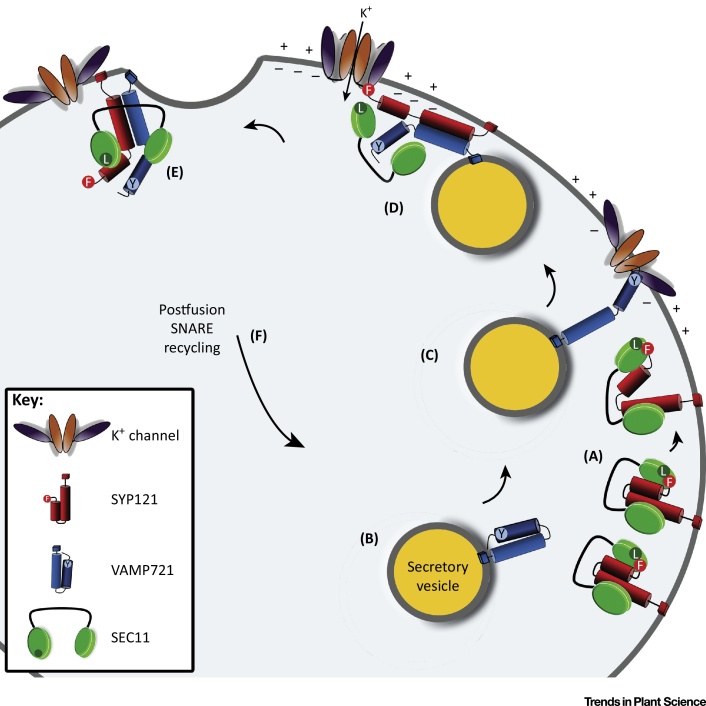
A Model for Kv Channel-Binding Exchange between VAMP721 and SYP121. K^+^ channels are shown with orange pore domains and purple voltage-sensor domains (VSDs). SYP121 is shown in red with membrane anchor, longer Qa-SNARE domain, and shorter Habc domain with the FxRF motif shown as a circle and Phe^9^ highlighted; VAMP721 is shown in blue with membrane anchor, longer R-SNARE domain, and shorter longin domain with Tyr^57^ indicated. SEC11 is depicted in green with the major cleft formed by the black line and residue Leu^128^, important for SEC11 interactions with SYP121 33, 88, is indicated near the N-terminal surface. Other SNARE proteins have been omitted for clarity. Voltage is denoted by charges (+ and –), with membrane hyperpolarisation indicated with increased negative charge inside the cell. (A) SYP121 in its ‘closed’ conformation is stabilised by SEC11 bound to the SYP121 N terminus with the interaction with Leu^128^ in the SEC11 minor cleft essential for unfurling of the SYP121 Habc domain. (B,C) VAMP721 on the secretory vesicle binds the closed K^+^ channel to help recruit the vesicle to the plasma membrane. (D) VAMP721 and the open conformation of SYP121 exchange channel binding, facilitated by membrane hyperpolarisation and channel opening with VSD in the ‘down’ state, assembling the *trans-*SNARE complex, which is stabilised by SEC11. (E) SEC11 facilitates release from channel and disassembly of the *cis*-SNARE complex. (F) SNARE complex disassembles to recycle the cognate SNAREs for subsequent fusion events. Abbreviation: SNARE, soluble *N*-ethylmaleimide-sensitive factor attachment protein receptor.

How, then, does SEC11 disengage from SYP121 to activate the Qa-SNARE? Similar to other SM proteins, SEC11 binding depends on the N terminus of the Qa-SNARE [94], and especially on a highly -conserved Phe^9^ residue present in SYP121 to promote secretory traffic [33]. Here, the Qa-SNARE interacts with the minor cleft and Leu^128^ near the N-terminal surface of SEC11 33, 88. Thus, the N-terminal site for SEC11 binding overlaps with the F^9^xRF motif that is essential for Kv channel binding [58]. Furthermore, only SYP121 and the SNARE complexes that it assembles will bind SEC11 at the plasma membrane 33, 88. It has been found that the channel-binding domain peptide KC1^Δ92-677^, which includes the RYxxWE motif, affects SEC11 binding to SYP121 as well as that of the cognate SNAREs in complex (S. Waghmare, R. Karnik and M.R. Blatt, unpublished, 2016), suggesting both a role for the Kv channel in SEC11 binding as well as a possible sequence involving the channel in a ‘handover’ between the Qa- and R-SNARE (Figure 4). In short, these findings are consistent with the idea that the Kv channel VSD is key to supporting secretory traffic *in vivo* through its action in promoting SYP121-mediated SNARE complex assembly.

## Concluding Remarks: A Century-Old Problem

Rates of cell expansion must be matched by solute transport and its accumulation as the cell volume increases. For the plant cell, it is vital that transport of inorganic solutes is maintained to provide osmotic pressure against the cell wall and, concurrently, that this process is controlled together with that of secretory traffic that delivers new membrane and cell wall material as the cell expands. Such coordination is intimately connected to mineral and water use by plants; its failure is associated with drought and salt sensitivities, with physiopathologies and diseases that reflect a loss in the control of cell volume and osmotic homeostasis, and with plant water relations and vegetative growth 67, 95, 96.

How widespread, then, are SNARE–channel interactions that might couple through membrane voltage in plants? Surveys of extant plant genomes 57, 60, 97 show that the F^9^xRF motif is closely conserved within a subset of plasma membrane Qa-SNAREs in vascular land plants, but not in bryophytes; neither is it found in animal Qa-SNAREs. The RYxxWE motif is similarly conserved within a subset of plant Kv channel VSDs in all vascular plant genomes [60], implying its early coevolution coincident with the expansion of SNAREs when plants colonised land [23]. Functional evidence to date is restricted to arabidopsis and tobacco, and there is much of SNARE protein function in plants that is still to be explored. Of interest, the same SNAREs also interact with a subset of plasma membrane-localised aquaporins 93, 98, suggesting functional controls that also extend to water flux rates across this membrane. It is likely that similar associations will surface in other vascular plants. What sets SNARE–-channel interactions in plants apart from the animal models [99] is the importance of regulating of membrane traffic and ion transport in parallel, with a basal mechanism of control through shared coupling to VSD conformation. Voltage is intimately connected with the osmotic solute uptake in plants. Its connection to membrane traffic and cell volume leaves little doubt now that this direct, mechanistic link lies at the heart of the century-old problem of how plants regulate turgor and cellular volume. From a practical point of view alone, understanding the mechanisms behind such coordination is bound to inform strategies for crop improvement in the face of global environmental change (see Outstanding Questions).

Outstanding Questions What is the sequence of binding and debinding events that leads to secretory vesicle fusion in association with K^+^ channel activation?

Does channel binding recruit SM protein binding or compete with it during SNARE complex assembly?

Do pathogens or symbionts take direct advantage of SNARE-channel interactions during their association with the plant host?

How might SNARE-channel interactions be manipulated to enhance growth, water, and mineral use efficiency by the plant?

## Figures and Tables

**Figure I:**
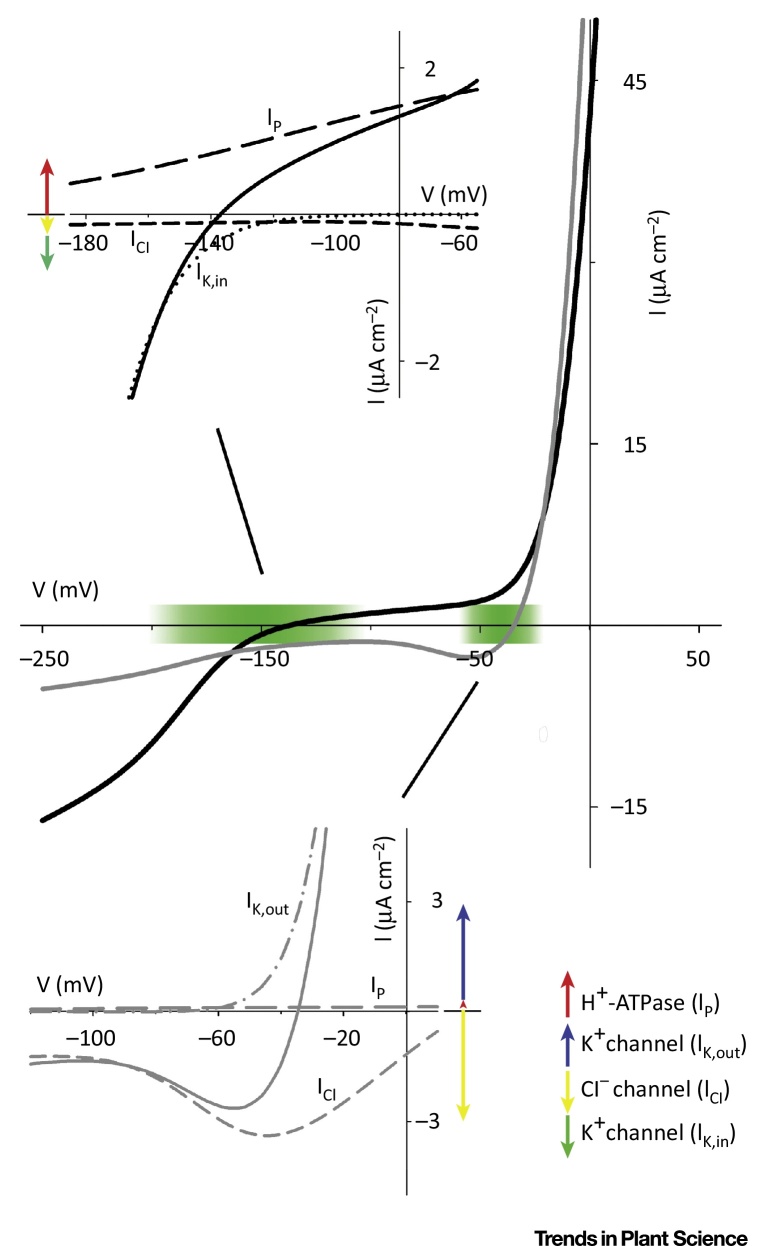
Total Membrane IV Curves and Their Decomposition.

## Box 1 Membrane Voltage Determines the Balance between Net Solute Uptake and Efflux

The membrane of plant cells typically exhibits two quasi-stable states, one hyperpolarised often to values negative of –100 mV and one depolarised and positive of the equilibrium voltage for K^+^ (E_K_). Membrane voltage when free running – not voltage clamped – is determined by the balance of current circulation through all charge-carrying transporters, such that the sum of all currents is always zero. Electrophysiologists commonly use current-voltage (IV) curves, such as the two shown in Figure I in the central panel (black, hyperpolarised; grey, depolarised), to describe current across the membrane. The IV curves shown were generated using OnGuard 49, 50, 104 and are typical of IV curves of arabidopsis guard cells and root epidermal cells. Experimentally, such IV curves are derived from measurements in which the membrane is clamped across a range of voltages and clamp current at each voltage is recorded [105]. Each curve is the sum of current through all transporters in the membrane. The point at which a curve crosses the voltage axis marks the free-running voltage; away from this point, there is either a net negative (inward) or positive (outward) current. Typical voltage ranges of the two quasi-stable states of the membrane are indicated by the green shading along the voltage axis.

The total membrane IV curve can be separated into the component IV curves for each transporter. Experimentally, separating transporter currents often relies on challenge with (ant-)agonists and, for voltage-gated channels, on the comparatively long timescales needed for channel gating to respond to changes in clamp voltage [105]. Each of the total membrane IV curves in Figure I is broken down to show the major components, namely the H^+^-ATPase (long-broken line), the inward- (dotted line) and outward-rectifying (broken-dotted line) K^+^ channel, and the Cl^–^ channel (short-broken line) currents. Current amplitudes and directions at the free-running voltage (where total membrane IV curve crosses the voltage axis) are indicated by the coloured arrows to the side in each case. Such analysis shows that several transporters are kinetically limited by voltage, notably the two K^+^ channels. It also shows that these transporters remain functional, even when their activity is constrained by voltage. K^+^ current through inward- (for uptake, primarily KAT1 in guard cells) and outward-rectifying (for efflux, primarily GORK) K^+^ channels is evident under voltage clamp, even when K^+^ is taken up during growth 55, 60, 106. Here, the outward-rectifying channels are evident as the substantial positive current near and positive of 0 mV in both of the total membrane IV curves. The main difference between the black and grey IV curves is a greater H^+^-ATPase and smaller Cl^–^ channel current in the hyperpolarised state (black IV curve). Also evident in this state is a larger inward-rectifying K^+^ channel current. These variations in transport capacity arise from post-translational regulation by cytosolic-free [Ca^2+^]. Thus, the dynamic range of voltages between the two states is dictated primarily by the two K^+^ channels and the voltage dependence of their activities.

## Glossary

Gating: a process of ion channel opening and closing, often responding to membrane voltage, ligand, or protein binding and debinding.
Kv channel: members of the gene superfamily of voltage-gated K^+^ channels that facilitate K^+^ flux across biological membranes; Kv channels assemble from four subunits, each with six transmembrane-spanning α-helices.
Sec1/Munc18 (SM) proteins: soluble proteins that bind SNAREs, regulating their assembly and stabilising the SNARE complex.
Soluble *N*-ethylmaleimide-sensitive factor attachment protein receptor (SNARE) proteins: membrane-anchored proteins that drive the final steps of membrane fusion.
*Trans*- and *cis*-SNARE complex: refers to the SNARE complex before fusion with proteins anchored in two membranes and after vesicle fusion with all SNARE proteins anchored in a single membrane, respectively.
V_1/2_: the voltage at which a population of voltage-gated ion channels achieves half-maximal activity; also known as the midpoint voltage, this parameter is a key descriptor of the gating process.
